# Impact of Predischarge NT-proBNP on Treatment Optimisation in Acute Heart Failure

**DOI:** 10.3390/ijms27021028

**Published:** 2026-01-20

**Authors:** Marija Polovina, Milenko Tomić, Milica Janković, Danka Civrić, Andrea Stojićević, Stefan Stanković, Teodora Pejović, Mihajlo Viduljević, Gordana Krljanac, Milika Ašanin, Sanja Stanković, Petar M. Seferović

**Affiliations:** 1 Faculty of Medicine, University of Belgrade, 11000 Belgrade, Serbia; gkrljanac@gmail.com (G.K.); masanin2013@gmail.com (M.A.); seferovic.petar@gmail.com (P.M.S.); 2 Department of Cardiology, University Clinical Centre of Serbia, 11000 Belgrade, Serbia; tomic_milenko@outlook.com (M.T.); jankovic.s.milica@gmail.com (M.J.); danka.civric@gmail.com (D.C.); andreastojicevic96@gmail.com (A.S.); stefan.stankovic019@gmail.com (S.S.); tea.pejovic3@gmail.com (T.P.); mihajloviduljevic@gmail.com (M.V.); 3 Faculty of Medical Sciences, University of Kragujevac, 34000 Kragujevac, Serbia; sanjast2013@gmail.com; 4 Center for Medical Biochemistry, University Clinical Centre of Serbia, 11000 Belgrade, Serbia; 5 Serbian Academy of Sciences and Arts, 11000 Belgrade, Serbia

**Keywords:** acute heart failure, heart failure with reduced ejection fraction, NT-proBNP, guideline-directed medical therapy, congestion, renal function

## Abstract

Residual congestion (RC) at discharge predicts adverse outcomes in heart failure with reduced ejection fraction (HFrEF). Its impact on the implementation of guideline-directed medical therapies (GDMT) remains unclear. N-terminal pro-B-type natriuretic peptide (NT-proBNP) trajectory during hospitalisation reflects RC and may be associated with GDMT implementation. The aim was to assess whether discharge NT-proBNP and a fall in NT-proBNP < 30% during hospitalisation (ΔNT-proBNP < 30%) predict GDMT underuse in acute HFrEF. In this prospective observational study, NT-proBNP was measured at hospital admission and 48–72 h before discharge. Provision of individual GDMT drug classes was assessed and GDMT underuse was defined as prescription of <3 key GDMT drug classes at discharge. 391 HFrEF patients (mean age, 69.9 ± 13.1years, 67.3% male) were included. ΔNT-proBNP < 30% was identified in 108 (27.6%). Higher discharge NT-proBNP was independently associated with lower likelihood of prescribing ACE-inhibitors, sacubitril/valsartan, eplerenone/spironolactone, or empagliflozin/dapagliflozin. ΔNT-proBNP < 30% was associated with 17% higher odds of GDMT underuse (95% confidence interval, 1.10–1.31, *p* < 0.001), regardless of clinical characteristics or in-hospital management. Patients with ΔNT-proBNP < 30% were discharged on lower doses of titratable GDMT medications. In-hospital NT-proBNP burden and trajectory, as markers of RC, are associated with GDMT underutilisation at discharge in acute HFrEF. Addressing RC may impact treatment quality in acute HFrEF.

## 1. Introduction

Acute heart failure (AHF) is a major cause of hospitalisation in Europe, exceeding 3.2 million admissions per year [1], and is followed by a high-risk period in which around 30% of patients are readmitted within 3 months postdischarge [2]. Importantly, a substantial proportion of patients (30.9%) leave the hospital with residual congestion [3], reflecting persistently elevated ventricular filling pressures despite medical therapy [4]. Multiple studies have shown a strong association between residual congestion and higher risks of rehospitalisation and mortality [3,4]. Natriuretic peptides (i.e., N-terminal pro-B-type natriuretic peptide, NT-pro-BNP) are markers of elevated ventricular filling pressures and their measurement is recommended at time of hospitalisation and before discharge for non-invasive assessment of the volume status and residual congestion [5]. Patients with AHF who achieve a fall in NT-proBNP levels ≥30% from admission to discharge have a significantly lower 6-month mortality compared to those with persistently elevated NT-proBNP levels [6]. However, a clinical trial, evaluating NT-proBNP-guided titration of guideline-directed medical therapy (GDMT) to reach NT-proBNP reduction ≥30% in patients with AHF, did not demonstrate improved 6-month outcomes [7]. Notably, GDMT changes in NT-proBNP-guided arm were modest [7]. In patients with AHF with reduced ejection fraction (HFrEF), there were no significant differences in the use of major HF drug classes, including angiotensin-converting enzyme inhibitors (ACEI) or angiotensin receptor blockers (ARB), mineralocorticoid receptor antagonists (MRA), and diuretics between treatment groups. Furthermore, beta-blockers were more frequently down-titrated in the NT-proBNP-guided group compared to conventional therapy [7]. These findings raise a possibility that therapeutic undertreatment, rather than the failure of NT-proBNP guidance per se, contributed to the neutral trial results. However, it remains unclear the extent to which residual congestion, assessed by natriuretic peptide levels, is associated with the underuse of GDMT. Therefore, the objective of the present study was to evaluate whether residual congestion, defined as persistently elevated discharge NT-proBNP levels and insufficient reduction in NT-proBNP during hospitalisation, is associated with GDMT underuse in acute HFrEF.

## 2. Results

### 2.1. Baseline Characteristics, Comorbidities, and Management During Hospitalisation

Between 27 December 2024 and 31 July 2025, we included a total of 391 patients (mean age, 69.9 ± 13.1years, 67.3% male) hospitalised for acute HFrEF and discharged alive. Of those patients, insufficient NT-proBNP reduction (ΔNT-proBNP < 30%) was identified in 108 patients (27.6%), whilst 283 (72.4%) patients had appropriate NT-proBNP reduction (∆NT-proBNP level ≥ 30%). The demographic and clinical characteristics of the study population are presented in Table 1. Patients with ΔNT-proBNP < 30% were older, more often had a history of heart failure (HF) rather than de novo HF, and had a higher burden of hypertension, coronary artery disease, and peripheral arterial disease. There were no differences in the prevalence of cardiomyopathy, atrial fibrillation, or prior stroke/transient ischaemic attack (TIA). The prevalence of comorbidities including a history of chronic kidney disease (CKD), anaemia, and type 2 diabetes mellitus (T2DM) was also higher in the ΔNT-proBNP < 30% group. No differences were observed in the prevalence of chronic obstructive pulmonary disease (COPD) or active smokers.

The clinical characteristics of the study groups are presented in Table 2. Patients with ΔNT-proBNP < 30% had lower admission systolic and diastolic blood pressure values, a higher baseline New York Heart Association (NYHA) functional class, and higher prevalence of pleural effusion at admission, albeit the prevalence of peripheral oedema was similar to patients with ∆NT-proBNP level ≥ 30%. Notably, patients with ΔNT-proBNP < 30% also had higher prevalence of predischarge pleural effusion and peripheral oedema. There was no difference in predischarge heart rate between the two groups, but bradycardia (discharge heart rate < 60 beats per minute) was less frequent in the ΔNT-proBNP < 30% group. Patients with ΔNT-proBNP < 30% had lower systolic and diastolic blood pressure values prior to discharge, with a higher prevalence of systolic blood pressure < 100 mmHg at discharge. Inotropic and/or vasopressor medications were more frequently used among patients with ΔNT-proBNP < 30%. Although there was no difference in the use of intravenous loop diuretics during the hospitalisation between the two groups, patients with ΔNT-proBNP < 30% required a higher peak loop diuretic dose, and more frequently needed the addition of a thiazide-like diuretic or oral acetazolamide. The length of the hospital stay was significantly longer in patients with insufficient NT-proBNP reduction (Table 2).

Laboratory findings, echocardiographic assessment, NT-proBNP levels at admission, and predischarge are summarised in Table 3. Patients with ΔNT-proBNP < 30% had higher baseline serum creatinine and blood urea nitrogen, as well as lower Na^+^ concentrations. They more often experienced worsening renal function and had higher incidences of hyperkalaemia during hospitalisation. The baseline and discharge levels of NT-proBNP were higher in patients with ΔNT-proBNP < 30% (Table 3).

### 2.2. Provision of Guideline-Directed Medical Therapies at Discharge

GDMT drugs prescribed at discharge are presented in Table 4. Discharge prescriptions reflect the cumulative continuation of prior therapy or de novo initiation of GDMT during hospitalisation following haemodynamic stabilisation. Patients with ΔNT-proBNP < 30% were less frequently prescribed with ACEI, sacubitril/valsartan, MRA, and SGLT2I, whereas there were no differences in the prescription of ARB and beta-blockers. All patients were discharged with loop diuretics. Notably, GDMT underutilisation (i.e., less than three GDMT drug classes) at discharge was significantly more frequent in patients with ΔNT-proBNP < 30% (Table 4).

**Figure 1:**
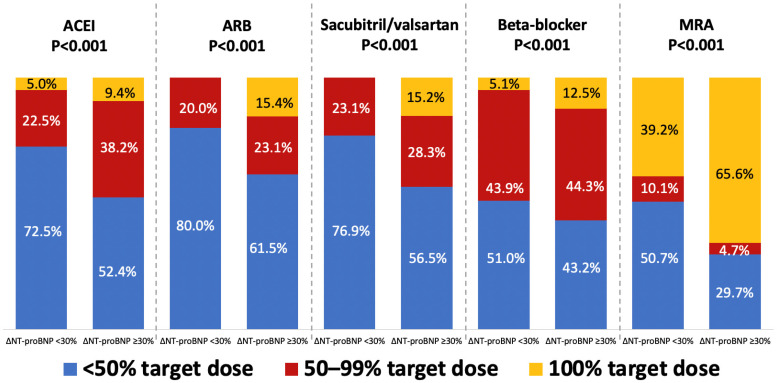
Provision of <50%, 50–99%, and 100% of the guideline-recommended target doses of titratable heart failure medications at discharge.

Regarding the dosing of GDMT medications, among patients discharged on titratable HF therapies (ACEI, ARB, sacubitril/valsartan, beta-blockers, and MRA), those with an insufficient reduction in NT-proBNP (ΔNT-proBNP < 30%) were more frequently discharged on lower medication doses, namely, <50% of the guideline-recommended target doses across all drug classes (Figure 1). Consistently, these patients were less likely to receive the optimal dosing of titratable GDMT (i.e., ≥50% of the target dose) at discharge (Figure 1).

### 2.3. Association Between Clinical Variables, Discharge NT-proBNP, and Key Guideline-Directed Medications for Heart Failure

The association between clinical variables and the provision of individual GDMT drug classes is presented in Supplementary Table S1. Variables with significant association with GDMT provision were used to construct multivariable regression model assessing the association between ln(discharge NT-proBNP) and the prescription of GDMT drug classes at discharge.

The association between discharge NT-proBNP levels, analysed as ln(discharge NT-proBNP), and the prescription of individual GDMT drug classes, as well as the overall extent of GDMT implementation, is illustrated in Table 5. For each 1 ln-unit increase in ln(discharge NT-proBNP), corresponding to a 2.7-fold increase in the original NT-proBNP value, the odds of being prescribed an ACEI, sacubitril/valsartan, MRA, or SGLT2I at discharge decreased by 35%, 43%, 28%, and 17%, respectively, after adjustment for covariates (Table 5).

After multivariable adjustment, each 1-unit increase in ln(discharge NT-proBNP) was associated with 19% higher odds of being provided with less than three GDMT drug classes at discharge (Table 5).

Furthermore, a multivariable logistic regression model, including clinically relevant variables and insufficient NT-proBNP reduction (ΔNT-proBNP < 30%), was used to assess the predictors of GDMT underutilisation (provision of less than three GDMT drug classes at discharge) (Table 6).

The multivariable analysis revealed that older age, lower NYHA class, low blood pressure at discharge, residual pleural effusion, hyperkalaemia, a history of CKD, worsening renal function during hospitalisation, and longer length of hospital stay, in addition to insufficient NT-proBNP reduction, predicted significant GDMT underutilisation at discharge. Higher LVEF was associated with a lower likelihood of GDMT underuse. Notably, in multivariable analysis, accounting for clinical factors, patients having insufficient NT-proBNP reduction (ΔNT-proBNP < 30%) had 17% higher odds of GDMT underuse (OR 1.17, 95% CI 1.10–1.31, *p* < 0.001).

Further interaction analyses revealed no significant interaction between insufficient NT-proBNP reduction and the presence of clinical signs of residual congestion at discharge, including pleural effusion (*p*-value for interaction = 0.411) or peripheral oedema (*p*-value for interaction = 0.288). There was also no significant interaction between ΔNT-proBNP < 30% and a history of CKD (*p*-value for interaction = 0.636) or worsening renal function during hospitalisation (*p*-value for interaction = 0.224).

## 3. Discussion

In this prospective study of 391 patients hospitalised for acute HFrEF, both higher discharge NT-proBNP levels and insufficient NT-proBNP reduction (ΔNT-proBNP < 30%) were independently associated with lower implementation of GDMT at discharge. Patients with insufficient NT-proBNP reduction were not only less likely to receive key HF medications during hospitalisation, but, also, when prescribed titratable GDMT drug classes, consistently received lower, suboptimal doses compared with patients achieving adequate NT-proBNP reduction. These findings suggest that residual congestion, as reflected by natriuretic peptide burden and trajectory, reflects clinical decisions regarding the initiation and intensification of disease-modifying therapies for HFrEF during hospitalisation.

Hospitalisation for HF is a critical event, associated with a significant risk of complications and adverse outcomes, whilst also providing an opportunity to optimise the treatment to reduce future risk of death, prevent readmissions, and improve quality of life [8]. A recent clinical trial of patients hospitalised for HF (68% with HFrEF) has demonstrated that timely provision of key GDMT medications (i.e., ACE/ARB or sacubitril/valsartan, beta-blockers, MRA) upon clinical stabilisation and before discharge can reduce the 6-month risk of rehospitalisation or all-cause mortality by 34% [9]. However, real-world data indicate that GDMT implementation in patients with HF remains suboptimal, particularly in clinically vulnerable subgroups, such as older, frail, or multimorbid individuals [10,11]. Earlier reports identified key clinical features, including older age and female sex, low blood pressure, bradycardia, hyperkalaemia, and CKD as major hurdles to GDMT implementation in real-world settings [12,13,14,15]. In addition, socioeconomic deprivation, longer duration of hospitalisation, and lower LVEF, as well as predischarge clinical evidence of congestion, were associated with significant GDMT underutilisation [16]. Residual congestion may reflect ongoing haemodynamic and neurohormonal instability, with increased susceptibility to hypotension, renal dysfunction, or hyperkalaemia, thereby limiting medication tolerance and supporting physicians’ reluctance to initiate and up-titrate GDMT medications [17].

In the present study, residual congestion, defined by persistently elevated NT-proBNP levels or failure to achieve ≥30% reduction during hospitalisation, was observed in 27.6% of patients, which is in line with earlier studies [3,7]. In contrast, clinical signs of congestion at discharge were less frequent, with pleural effusion and peripheral oedema evident in 13.0% and 23.8% of patients, respectively. Patients with persistent congestion represented a particularly vulnerable subgroup, characterised by older age, longer duration of HF, a higher prevalence of CV and non-CV comorbidities, and more severe clinical presentation and symptom burden. They more frequently experienced a complicated clinical course (e.g., worsening renal function and hyperkalaemia) and required inotropic or vasopressor support, as well as more intensive decongestive therapies. Consistent with this profile, these patients also more frequently exhibited clinical evidence of residual volume overload, including predischarge pleural effusion and peripheral oedema. They more frequently exhibited low blood pressure at discharge (i.e., systolic blood pressure < 100 mmHg), a well-recognised cause of treatment intolerance and an important barrier to the successful implementation of many HF therapies [18]. Despite a longer duration of hospitalisation, their clinical profiles and overall in-hospital courses were more complex, which limited the appropriate initiation and optimisation of GDMT. Current evidence demonstrates that early implementation of GDMT, once haemodynamic and clinical stability is achieved and prior to hospital discharge, confers substantial prognostic benefit, with further dose optimisation in the weeks following discharge [9]. Collectively, these findings underscore that not only GDMT use per se, but also its timely initiation, is critical for improving outcomes—a concept strongly emphasised in current clinical guidelines [5].

The overall GDMT provision in the present study was high, with 61.2% of patients receiving an ACEI/ARB, additional 28.6% sacubitril/valsartan, 93.0% a beta-blocker, 76.9% an MRA, and 64.4% an SGLT2I. However, most patients, especially those with insufficient NT-proBNP reduction (ΔNT-proBNP < 30%), were discharged on <50% of the guideline-recommended target doses of titratable HF therapies, likely reflecting ongoing efforts toward dose optimisation. These findings align with recent European data indicating progressive improvement in GDMT uptake and optimisation before hospital discharge [10,16]. However, individuals with insufficient NT-proBNP reduction (∆NT-proBNP < 30%) less frequently received all classes of GDMT, except ARB (likely due to a small number of treated patients) and beta-blockers. The suboptimal dosing of HF therapies was more common in this group, particularly with respect to ACEI/ARB and sacubitril/valsartan. Furthermore, insufficient predischarge NT-proBNP reduction (∆NT-proBNP < 30%) was associated with 17% higher odds of GDMT underuse, defined as the prescription of fewer than three fundamental GDMT drug classes at discharge. Our findings confirmed previous observations that older age, lower LVEF, more severe symptom burden, lower blood pressure at discharge, persisting clinical congestion, a history of CKD or worsening renal function, and longer hospital stays, also predict GDMT underutilisation in HFrEF patients. These features reflect the more advanced disease, greater multimorbidity and complicated clinical course, and greater propensity for GDMT intolerance or adverse effects. Importantly, the association between ∆NT-proBNP < 30% and drug underuse was independent of these clinical characteristics and persisting congestion. This suggests that NT-proBNP trajectory reflects clinical instability not fully captured by bedside assessment, which may reflect treatment decisions beyond overt congestion at discharge. Reliance solely on clinical signs of congestion, which may be subtle and easily overlooked in clinical practice, particularly when intravascular rather than interstitial congestion predominates, may underestimate the true burden of residual volume overload, as reflected by persistently elevated natriuretic peptide levels [19,20]. Indeed, insufficient reduction in NT-proBNP levels was associated with lower utilisation of key neurohormonal axis inhibitors (i.e., ACEI, sacubitril/valsartan, and MRA), where concerns of intolerance influenced by haemodynamic factors may have underlined therapeutic reluctance [17]. However, persistently higher NT-proBNP levels were also associated with lower prescription of SGLT2I, which have negligible haemodynamic effects, indicating other contributing factors. Furthermore, the association between insufficient NT-proBNP reduction and GDMT underuse was consistent regardless of CKD, worsening renal function, hyperkalaemia, or the intensity of diuretic treatment during hospitalisation. This challenges the common perception that renal dysfunction, electrolyte disturbances, or insufficient diuretic treatment drive GDMT withholding. These results also confirm the benefit of natriuretic peptide testing in AHF patients with CKD or worsening renal function, where test interpretation may be confounded by renal impairment [21,22]. However, the association between persistently elevated NT-proBNP levels and GDMT underuse should not be interpreted as a causal relationship. Rather, the sustained elevation of natriuretic peptides, reflecting ongoing congestion, likely serves as a marker of more advanced disease and broader clinical instability not fully captured by the variables included in the present analysis.

Importantly, failure to initiate GDMT may itself contribute to persistently elevated natriuretic peptide levels, suggesting a bidirectional relationship between effective neurohormonal blockade and SGLT2I therapy, and successful decongestion, as reflected by lower biomarker concentrations. Evidence from a clinical trial demonstrated that early and intensive in-hospital initiation of neurohormonal inhibitors (ACEI or ARB or sacubitril/valsartan, beta-blockers, and MRA) in stabilised patients with AHF resulted in more pronounced and sustained decongestion, accompanied by lower predischarge NT-proBNP levels compared with standard care [23]. Moreover, in patients hospitalised for AHF, treatment with the SGLT2I empagliflozin was associated with a greater reduction in NT-proBNP during hospitalisation than placebo [24].

The present findings support the value of natriuretic peptide assessment at admission and before discharge as a part of a multiparametric approach to evaluate (de)congestion, by integrating clinical, laboratory, and imaging findings during hospitalisation [19]. This integrative approach should incorporate several assessment tools, including follow-up lung ultrasound to evaluate the regression of B-lines or repeat chest radiography, serial measurements of inferior vena cava diameter and collapsibility, obtaining a Venous Excess Ultrasound (VExUS) score to assess residual venous, liver, and kidney congestion, and/or the use of additional congestion biomarkers, such as changes in haematocrit or carbohydrate antigen 125 (CA-125) levels [19]. These measures may provide additive prognostic value beyond clinical findings and natriuretic peptides, and offer greater sensitivity for specific HF phenotypes, including right-sided HF [22]. They can help identify patients with persisting congestion, who may require more intensive efforts with diuretic combinations, and natriuresis monitoring to obtain euvolemia during hospitalisation [25,26]. This may favourably reflect on decreasing the number of patients being discharged with residual volume overload, who later have higher risks of mortality and readmissions [3]. These results also support the importance of GDMT optimisation, even in individuals with residual congestion. Indeed, a clinical trial provides support that patients with insufficient NT-proBNP decline (i.e., less than 10% from baseline levels, but still >1500 pg/mL) can successfully tolerate intensive GDMT initiation and rapid up-titration before discharge [9]. Further analysis of this trial confirmed that intensive GDMT optimisation in clinically stable, but modestly congested, individuals, can provide more sustained decongestion over time, ultimately translating into improved outcomes [23]. The present findings extend these observations by suggesting that achieving clinical stability should prompt, rather than delay, GDMT implementation in acute HFrEF, even in patients with persistently elevated NT-proBNP levels. As previously demonstrated, incorporating NT-proBNP trajectory can facilitate therapeutic decisions [27]. NT-proBNP should be preferred over B-type natriuretic peptide (BNP), as its circulating levels (unlike those of BNP) are not affected by neprilysin inhibition with sacubitril/valsartan, which can lead to artificially elevated BNP concentrations [28]. Early initiation of therapies with favourable haemodynamic profiles and the ability to promote natriuretic peptide decline, such as SGLT2I and sacubitril/valsartan, may be particularly beneficial [29,30]. By targeting volume overload alone and potentially exacerbating neurohormonal activation, diuretics do not adequately address the underlying pathophysiological mechanisms driving congestion and HF progression [31]. By contrast, early GDMT initiation can act synergistically with diuretics to attenuate detrimental neurohormonal activation, thereby promoting more sustained decongestion and facilitating more effective implementation of disease-modifying therapies [31].

### Study Limitations

Several limitations need to be considered regarding the present study. This was a single-centre study with a relatively small sample size, which may limit generalisability; therefore, the findings should be confirmed in larger, multicentre studies. Nevertheless, the observed clinical characteristics and high GDMT uptake are consistent with recent European data, supporting external relevance [10,16]. The observed association between persistently elevated biomarkers of congestion and other clinical variables identified to predict lower GDMT uptake should not be interpreted as causal. Given the observational design of the study, these findings are hypothesis generating and warrant further evaluation in appropriately designed interventional trials. GDMT underuse was defined as the prescription of fewer than three GDMT drug classes at discharge, in accordance with limited prior clinical trial and observational data, although other definitions may be applicable. Only patients with HFrEF were included; therefore, the findings may not be applicable to patients with different HF phenotypes, in whom NT-proBNP values and dynamics may be different [22]. This is particularly relevant for patients with obesity and HF with preserved ejection fraction and for those with predominant right-sided HF, who may exhibit “lower-than-expected” NT-proBNP levels due to increased clearance in adipose tissue or smaller myocardial mass despite clear evidence of congestion [32,33]. Residual congestion was assessed using NT-proBNP trajectory and clinical signs (pleural effusion and peripheral oedema). More advanced tools for congestion assessment, including lung ultrasound, venous ultrasound (VExUS), invasive haemodynamic assessment, or serial weight and natriuresis measurements, were not available and could have provided complementary information. NT-proBNP was measured at admission and 48–72 h prior to discharge. Intermediate measurements were not analysed, and, therefore, dynamic changes during hospitalisation may not have been fully captured, which is possibly relevant for the clinical course and treatment of the patients. In addition, NT-proBNP levels may be influenced by factors beyond congestion, including renal function, obesity, age, and lack of GDMT implementation, although these were adjusted for in multivariable models. Although no significant interactions were observed between insufficient NT-proBNP reduction and renal function or clinical congestion, the study may have been underpowered, and these findings should be interpreted cautiously. The analysis was limited to GDMT prescription at discharge and did not evaluate (dis)continuation of prior therapy, timing of GDMT initiation during hospitalisation, or whether initial underutilisation persisted over time and possibly translated into adverse postdischarge clinical outcomes.

## 4. Materials and Methods

### 4.1. Study Design and Inclusion Criteria

This was an observational, prospective study including patients admitted for AHF and discharged alive, between December 2024 and July 2025, from the Department of Cardiology at the University Clinical Centre of Serbia, Belgrade, Serbia, with a primary diagnosis of HF. Only patients with HFrEF, based on echocardiographically documented LVEF ≤ 40% during the hospital stay were included [5]. The following exclusion criteria were applied: (1) cardiogenic shock; (2) advanced HF (as per published criteria) and documented GDMT intolerance [5,34]; (3) severe CKD defined as persistently decreased eGFR < 30 mL/min/1.73 m^2^ according to the published criteria, due to limited supporting recommendations for the use of most GDMT medications and their impact on the interpretation of natriuretic peptide testing [5,35,36]; (4) inability to provide informed consent. The study protocol was approved by the Institutional Review Board and the Ethics Committee of the University Clinical Centre of Serbia (No 420/1) and the consent form for participation was distributed to all participants and signed.

### 4.2. Data Collection and Assessment of NT-proBNP Changes During the Hospital Stay

Anonymised data on patients’ demographic characteristics (age, sex), clinical presentation (heart rate, systolic and diastolic blood pressure), signs of congestion at admission and predischarge, and admission New York Heart Association (NYHA) class were collected. Data were collected on the use and the peak daily dose of intravenous loop diuretics (e.g., furosemide) during hospitalisation and the concomitant use of thiazide-type diuretics or oral acetazolamide for decongestion. Further, information on the use of inotropic and/or vasopressor medications was obtained, including intravenous catecholamine agents, milrinone, and/or levosimendan. Data were extracted from medical records on standard transthoracic echocardiography and routine laboratory findings. Serum K^+^ and creatinine levels were collected throughout hospitalisation. Hyperkalaemia was defined as serum K^+^ > 5.5 mmol/L, whilst worsening renal function was defined as serum creatinine increase ≥26.5 μmol/L within 72 h [37]. We also collected data on HF (previously know or de novo) and the following CV comorbidities: arterial hypertension (systolic/diastolic blood pressure > 140/90 mmHg and/or antihypertensive treatment), ischaemic heart disease (known angina pectoris, previous myocardial infarction, and/or percutaneous or surgical coronary revascularisation), cardiomyopathy (dilated or other with HFrEF), atrial fibrillation, stroke/transient ischaemic attack (TIA) and peripheral vascular disease. Non-CV comorbidities were assessed including T2DM (previous diagnosis and/or treatment with glucose-lowering medications or T2DM diagnosed during hospitalisation), CKD (glomerular filtration rate estimated with CKD-EPI equation, eGFR ≥ 30 to <60 mL/min/1.73m^2^, documented 3 months before hospitalisation or persistently decreased eGFR ≥ 30 to <60 mL/min/1.73m^2^ during hospitalisation), COPD (according to medical records), and anaemia (haemoglobin < 130 g/L in men, <120 g/L in women). Blood samples for NT-proBNP analysis were collected at the time of admission and subsequently 48–72 h before discharge. NT-proBNP levels were measured using a commercial electrochemiluminescence immunoassay (ECLIA) kit on the Cobas^®^ e801 immunoassay analyser (Roche Diagnostics GmbH, Mannheim, Germany). NT-proBNP concentrations were expressed in pg/mL. The study population was divided into two groups: (1) patients having predischarge reduction in NT-proBNP <30% compared to admission levels (i.e., ∆NT-proBNP < 30%) value were considered to have laboratory evidence or residual congestion; (2) patients having predischarge NT-proBNP reduction >30% compared to admission values (i.e., ∆NT-proBNP level ≥ 30%) were considered to have appropriate NT-proBNP reduction.

### 4.3. Prescription of Guideline-Directed Medical Therapy for Heart Failure at Discharge

From the day of admission, HF treatment was started or continued at the discretion of the treating physician until discharge. Data were collected at discharge for each included patient on the prescription of the four fundamental GDMT drug classes for HFrEF—ACEI or ARB or sacubitril/valsartan, beta-blockers, MRA (spironolactone or eplerenone), and SGLT2I (dapagliflozin or empagliflozin)—as well as the use of loop diuretics at discharge [5]. GDMT underutilisation was defined as prescription of less than three fundamental GDMT drug classes at discharge. This definition was selected based on evidence from a previous clinical trial in AHF patients demonstrating improved outcomes with the prescription of at least three key HF drug classes at discharge, as well as on consistency with previous observational studies [9,16]. Data were additionally collected on the prescribed doses of GDMT drug classes requiring up-titration to guideline-recommended target doses (ACE, ARB, sacubitril/valsartan, beta-blockers, and MRA). Based on prior evidence indicating that attainment of at least 50% of the target dose of titratable GDMT medications is associated with clinical benefits comparable to those of full target dosing [38], doses were categorised as <50% of the target dose, 50–99% of the target dose, or 100% of the target dose.

### 4.4. Statistical Analysis

Since this was an observational study, no formal sample size calculation was done. Continuous variables are presented as means and standard deviations or medians and interquartile range, as suitable, whilst categorical data were presented as frequencies with percentages. Between-group differences were assessed using an independent-samples *t*-test or the Mann–Whitney U test for continuous variables, and the χ^2^ test for categorical variables, as appropriate. The association between discharge NT-proBNP levels and the prescription of each individual GDMT drug class (ACEI, ARB, sacubitril/valsartan, beta-blocker, an MRA, and a SGLT2I) was assessed using binary logistic regression. The association between discharge NT-proBNP levels and GDMT underuse, expressed as provision of fewer than three fundamental GDMT classes, was also evaluated using binary logistic regression. Discharge NT-proBNP concentrations were log-transformed using the natural logarithm prior to analysis due to right-skewed distributions. The association between clinically relevant covariates and GDMT underuse was assessed using binary logistic regression. These variables were selected a priori based on biological plausibility and existing evidence [16], including age, sex, LVEF, NYHA class III-IV, clinical signs of residual congestion at discharge (pleural effusion and peripheral oedema), hyperkalaemia, history of CKD, worsening renal function during hospitalisation, peak daily dose of intravenous diuretic, addition of thiazide-type diuretic or oral acetazolamide during hospitalisation, use of inotropes/vasopressors, low blood pressure at discharge (systolic blood pressure at discharge < 100 mmHg), bradycardia (discharge heart rate < 60 beats per minute), and length of hospital stay. Variables showing a significant association (*p* < 0.05) in univariable analyses were used to adjust the multivariable models on the association of ln (discharge NT-proBNP) and the use of individual GDMT drug classes as well as the overall GDMT underuse. In addition, the association between ΔNT-proBNP < 30% and GDMT underutilisation at discharge, i.e., prescription of fewer than three fundamental GDMT classes, was also evaluated by constructing a multivariable logistic regression model including ΔNT-proBNP < 30% and those clinical covariates with a significant association with GDMT underuse (*p* < 0.05). Pre-specified interaction terms between ΔNT-proBNP < 30% and predischarge pleural effusion (ΔNT-proBNP < 30% * x predischarge pleural effusion), predischarge peripheral oedema (ΔNT-proBNP < 30% x predischarge peripheral oedema), a history of CKD (ΔNT-proBNP < 30% x CKD), and worsening renal function during hospitalisation (ΔNT-proBNP < 30% x serum creatinine increase ≥ 26.5 μmol/L/L over 72 h), were additionally included in the model. All analyses were performed using IBM SPSS Statistics (version 30). Statistical significance was defined as a two-sided *p* value < 0.05.

## 5. Conclusions

In patients hospitalised for acute HFrEF, residual congestion, reflected by persistently elevated discharge NT-proBNP levels and insufficient NT-proBNP reduction (i.e., ΔNT-proBNP < 30%) during hospitalisation was associated with suboptimal GDMT utilisation and dosing at discharge. This association was consistent regardless of overt clinical signs of congestion, renal dysfunction, or other characteristics and in-hospital management, suggesting that natriuretic peptide dynamics capture a clinically significant dimension that contributes to reluctance to initiate optimal medical therapy in patients with HFrEF. These findings highlight the importance of integrating NT-proBNP trajectory into a comprehensive assessment of decongestion. They underscore the need for improved clinician education on the interpretation and clinical integration of natriuretic peptide dynamics, as well as for early implementation of GDMT to facilitate more effective decongestion and improved outcomes [39]. Addressing residual congestion before discharge may reduce therapeutic inertia and enhance the quality of care in acute HFrEF.

## Figures and Tables

**Table 1:** Demographic characteristics and comorbidities.

Variable	All Patients, n = 391	∆NT-proBNP < 30%, n = 108 (27.6%)	∆NT-proBNP ≥ 30%, n = 283 (72.4%)	*p*-Value
Age (years)	69.9 ± 13.1	69.0 ± 12.6	65.4 ± 13.3	0.009
Sex (male), n (%)	263 (67.3)	70 (64.8)	193 (68.5)	0.675
Cardiovascular history				
Previous HF, n (%)	223 (57.0)	72 (66.7)	151 (53.4)	0.009
De novo HF, n (%)	168 (43.0)	36 (33.3)	132 (46.6)	0.009
Hypertension, n (%)	325 (83.1)	98 (90.7)	227 (80.2)	0.005
Coronary artery disease, n (%)	155 (39.6)	52 (48.1)	103 (36.4)	0.020
Cardiomyopathy, n (%)	156 (39.9)	47 (43.5)	109 (38.5)	0.372
Atrial fibrillation, n (%)	140 (35.8)	41 (37.9)	99 (34.9)	0.850
Stroke/TIA, n (%)	35 (8.9)	13 (12.0)	22 (7.8)	0.159
Peripheral arterial disease, n (%)	51 (13.0)	26 (24.1)	25 (8.8)	<0.001
Non-cardiovascular comorbidities				
CKD, n (%)	147 (37.5)	53 (49.1)	94 (33.2)	<0.001
Anaemia, n (%)	56 (14.3)	18 (16.7)	28 (9.9)	0.015
Type 2 diabetes, n (%)	125 (32.0)	45 (41.7)	80 (28.3)	0.015
COPD, n (%)	53 (13.6)	15 (13.9)	38 (13.4)	0.750
Active smoker, n (%)	71 (18.1)	22 (20.4)	49 (17.3)	0.466

sCr—serum creatinine, CKD—chronic kidney disease, COPD—chronic obstructive pulmonary disease, HF—heart failure, NYHA—New York Heart Association, TIA—transient ischaemic attack.

**Table 2:** Clinical characteristics and management during hospitalisation.

Clinical Features	All Patients, n = 391	∆NT-proBNP < 30%, n = 108 (27.6%)	∆NT-proBNP ≥ 30%, n = 283 (72.4%)	*p*-Value
Heart rate at admission (bpm)	92.3 ± 23.9	92.0 ± 24.3	93.2 ± 23.7	0.619
Heart rate at discharge (bpm)	82.3 ± 20.3	81.4 ± 18.6	83.5 ± 21.1	0.854
Discharge heart rate < 60 bpm, n (%)	43 (10.9)	8 (7.4)	35 (12.4)	0.068
Systolic BP at admission (mmHg)	122.5 ± 25.2	118.3 ± 24.4	125.4 ± 25.2	0.006
Systolic BP at discharge (mmHg)	115.8 ± 21.6	113.2 ± 21.2	117.4 ± 22.3	0.001
Diastolic BP (mmHg)	76.1 ± 15.6	72.9 ± 16.4	78.3 ± 14.6	<0.001
Diastolic BP at discharge (mmHg)	75.3 ± 12.1	72.7 ± 12.8	77.2 ± 13.9	0.003
Systolic BP at discharge < 100 mmHg, n (%)	27 (7.9)	10 (9.2)	17 (6.0)	<0.001
NYHA class II, n (%)	236 (60.3)	52 (48.1)	184 (65.0)	<0.001
NYHA class III-IV, n (%)	155 (39.7)	56 (51.9)	99 (35.0)	<0.001
Pleural effusion at admission, n (%)	168 (43.0)	58 (53.7)	110 (39.0)	0.011
Pleural effusion at discharge, n (%)	51 (13.0)	30 (27.8)	21 (8.8)	<0.001
Peripheral oedema at admission, n (%)	285 (73.0)	84 (77.8)	201 (71.0)	0.331
Peripheral oedema at discharge, n (%)	93 (23.8)	30 (27.8)	63 (22.2)	<0.001
Inotropes/vasopressors use, n (%)	55 (14.1)	22 (20.4)	33 (11.7)	<0.001
Intravenous loop diuretic use, n (%)	391 (100)	108 (100)	283 (100)	NA
Peak daily intravenous diuretic dose (mg/day), n (%)	225 ± 115	240 ± 120	160 ± 100	0.021
Use of thiazide-type diuretics or acetazolamide, n (%)	81 (20.7)	32 (29.6)	49 (17.3)	0.014
Length of hospital stay (days)	9.3 (5.7)	11.2 (5.3)	8.1 (3.9)	<0.001

BP—blood pressure, bpm—beats per minute, NYHA—New York Heart Association.

**Table 3:** Laboratory and echocardiographic findings.

Variable	All Patients, n = 391	∆NT-proBNP < 30%, n = 108 (27.6%)	∆NT-proBNP ≥ 30%, n = 283 (72.4%)	*p*-Value
Laboratory assessment				
Admission sCr (μmol/L)	91.5 ± 18.4	103.3 ± 32.0	85.6 ± 17.8	<0.001
Admission blood urea nitrogen (mmol/L)	6.7 ± 4.5	7.2 ± 5.3	5.4 ± 2.2	0.004
sCr increase ≥ 26.5 μmol/L/L over 72 h, n (%)	62 (15.8)	26 (24.1)	36 (12.7)	<0.001
Na^+^ (mmol/L)	138.9 ± 8.6	137.9 ± 13.1	139.6 ± 3.6	0.009
Na^+^ < 135 mmol/L during hospitalisation	68 (17.4)	33 (20.7)	35 (15.1)	0.113
K^+^ (mmol/L)	4.3 ± 0.7	4.4 ± 0.8	4.2 ± 0.5	0.009
Hyperkalaemia, n (%) *	40 (10.2)	22 (20.3)	18 (6.3)	<0.001
Haemoglobin (g/L)	133.9 ± 20.9	129 ± 23.2	135.2 ± 19.0	0.015
Maximum hsTn-T (ng/mL)	75 (25–93)	78 (31–100)	71 (22–90)	0.065
Admission NT-proBNP (pg/mL)	6631 (2223–15,221)	8855 (3246–18,326)	5751 (2128–13,952)	<0.001
Discharge NT-proBNP (pg/mL)	3931 (792–8986)	6321 (1488–12,566)	1451 (512–3224)	<0.001
Echocardiographic assessment				
LVEF (%)	28.9 ± 8.8	26.1 ± 9.3	31.1 ± 8.3	0.002
Moderate/severe mitral regurgitation, n (%)	123 (31.5)	45 (41.7)	78 (32.8)	0.023
Aortic stenosis, n (%)	47 (12.0)	15 (13.9)	32 (11.3)	0.853

hsTnT—high sensitivity troponin T, LVEF—left ventricular ejection fraction, NT-proBNP—N-terminal pro-B type natriuretic peptide * Hyperkalaemia—peak serum K^+^ during hospitalisation > 5.5 mmol/L.

**Table 4:** Medical therapy for heart failure at discharge.

Variable	All Patients, n = 391	∆NT-proBNP < 30%, n = 108 (27.6%)	∆NT-proBNP ≥ 30%, n = 283 (72.4%)	*p*-Value
ACEI, n (%)	221 (56.5)	40 (37.0)	181 (63.9)	<0.001
ARB, n (%)	18 (4.6)	5 (4.6)	13 (4.6)	0.786
Sacubitril/valsartan, n (%)	112 (28.6)	13 (12.0)	99 (34.9)	<0.001
Beta-blocker, n (%)	364 (93.0)	98 (90.7)	266 (94.0)	0.178
MRA, n (%)	301 (76.9)	69 (63.4)	232 (81.9)	0.013
SGLT2I, n (%)	252 (64.4)	51 (47.2)	201 (71.0)	<0.001
Loop diuretic, n (%)	391 (100)	108 (100)	283 (100)	NA
Furosemide-equivalent oral loop diuretic dose (mg)	60 ± 40	80 ± 45	40 ± 20	<0.001
Less than three key drug classes, n (%)	103 (26.3)	48 (44.4)	55 (19.4)	<0.001

ACEI—angiotensin-converting enzyme inhibitor, ARB—angiotensin receptor blocker, MRA—mineralocorticoid receptor antagonist, SGLT2I—sodium-glucose cotransporter-2 inhibitor.

**Table 5:** Association between ln(discharge NT-proBNP) and prescription of GDMT drugs.

Variable	Adjusted OR * (95% CI)	*p* Value
ACEI	0.65 (0.51–0.81)	<0.001
ARB	0.94 (0.56–1.27)	0.326
Sacubitril/valsartan	0.57 (0.33–0.75)	<0.001
Beta-blocker	0.86 (0.61–1.11)	0.178
MRA	0.72 (0.48–0.92)	<0.001
SGLT2I	0.83 (0.55–0.94)	<0.001
Less than three key drug classes	1.19 (1.03–1.55)	<0.001

ACEI—angiotensin-converting enzyme inhibitor, ARB—angiotensin receptor blocker, CI—confidence interval, MRA—mineralocorticoid receptor antagonist, OR—odds ratio, SGLT2I—sodium-glucose cotransporter-2 inhibitor. * Adjusted for: age, sex, LVEF, NYHA class, clinical signs of residual congestion at discharge (pleural effusion and peripheral oedema), discharge heart rate < 60 beats per minute, discharge systolic blood pressure at discharge < 100 mmHg), hyperkalaemia (serum K^+^ > 5.5 during hospitalisation), history of CKD, worsening renal function during hospitalisation (serum creatinine increase ≥ 26.5 μmol/L within 72 h), use of inotropes and/or vasopressors, peak daily dose of intravenous diuretic, addition of thiazide-type diuretic or oral acetazolamide during hospitalisation, and length of hospital stay.

**Table 6:** Association between clinical variables and insufficient NT-proBNP reduction and provision of less than three key GDMT drug classes.

Variable	Univariable OR (95% CI)	Multivariable OR (95% CI)	*p* Value
Sex (male)	0.98 (0.91–1.12)	0.99 (0.92–1.11)	0.246
Age (per year)	1.07 (1.02–1.10)	1.06 (1.02–1.09)	<0.001
LVEF (%)	0.87 (0.64–0.91)	0.85 (0.67–0.90)	<0.001
NYHA class III-IV	1.15 (1.10–1.25)	1.13 (1.09–1.26)	0.015
Discharge heart rate < 60 bpm	1.33 (1.22–1.56)	1.23 (0.99–1.49)	0.067
Discharge systolic BP < 100 mmHg	2.21 (1.88–2.78)	2.19 (2.01–2.57)	<0.001
Pleural effusion at discharge	1.23 (1.11–1.34)	1.16 (1.03–1.37)	<0.001
Peripheral oedema at discharge	1.16 (1.08–1.34)	1.12 (1.00–1.45)	0.088
Hyperkalaemia	1.69 (1.23–2.00)	1.71 (1.30–1.99)	<0.001
History of CKD	2.11 (1.78–3.10)	2.13 (1.81–2.89)	<0.001
Worsening renal function during hospitalisation	2.78 (2.23–3.21)	2.89 (2.51–2.97)	<0.001
Inotropes/vasopressors use	1.15 (1.02–0.28)	1.08 (0.98–1.17)	0.433
Peak daily dose of intravenous diuretic	1.09 (1.00–1.14)	1.05 (0.88–1.10)	0.771
Addition of thiazide-type diuretic or oral acetazolamide during hospitalisation	0.86 (0.80–0.94)	0.86 (0.81–1.02)	0.098
Length of hospital stay	1.07 (1.02–1.18)	1.07 (1.02–1.16)	<0.001
ΔNT-proBNP < 30%	1.21 (1.10–1.38)	1.17 (1.10–1.31)	<0.001

BP—blood pressure, CI—confidence interval, CKD—chronic kidney disease, LVEF—left ventricular ejection fraction, NYHA—New York Heart Association, ΔNT-proBNP < 30%—NT-proBNP reduction < 30% during hospitalisation.

## Data Availability

The data presented in this study are available on request from the corresponding authors due to privacy and ethical restrictions implied by the Ethics Committee of the University Clinical Centre of Serbia.

## Supplementary Materials

The following supporting information can be downloaded at: https://www.mdpi.com/article/10.3390/ijms27021028/s1.
