# Supplementary material for: Impact of Predischarge NT-proBNP on Treatment Optimisation in Acute Heart Failure
Source: Int J Mol Sci. 2026 Jan 20;27(2):1028. doi: 10.3390/ijms27021028 (PMC12842275; doi:10.3390/ijms27021028)
Supplement: Supplementary file 1 [file ijms-27-01028-s001.zip › ijms-4084466-supplementary.pdf]

Supplementary Table S1. Univariable logistic regression for the association between clinical variables and provision of the guideline-directed medical therapies at discharge

Variable	OR (95% CI)	*p* Value
**ACEI**		
Sex (male)	1.02 (0.87 – 1.11)	0.567
Age (per year)	0.95 (0.91 – 0.97)	0.010
LVEF (%)	1.11 (1.07 – 1.18)	<0.001
NYHA class III-IV	0.86 (0.78 – 1.08)	0.178
Discharge heart rate <60 bpm	0.92 (0.78 – 1.16)	0.279
Discharge systolic BP <100 mmHg	0.75 (0.58 – 0.88)	<0.001
Pleural effusion at discharge	0.78 (0.67 – 0.98)	<0.001
Peripheral oedema at discharge	0.80 (0.68 – 0.91)	<0.001
Hyperkalaemia	0.75 (0.55 – 0.85)	<0.001
History of CKD	0.83 (0.76 – 0.91)	<0.001
Worsening renal function during hospitalisation	0.56 (0.34 – 0.78)	<0.001
Inotropes/vasopressors use	0.79 (0.58 – 0.92)	<0.001
Peak daily dose of intravenous diuretic	0.88 (0.77 – 1.13)	0.067
Addition of thiazide-type diuretic or oral acetazolamide during hospitalisation	1.11 (0.99 – 1.58)	0.085
Length of hospital stay	1.07 (1.02 – 1.18)	<0.001
**ARB**		
Sex (male)	1.02 (0.89 – 1.09)	0.869
Age (per year)	0.94 (0.71 – 1.19)	0.117
LVEF (%)	1.12 (1.01 – 1.37)	<0.001
NYHA class III-IV	0.91 (0.54 – 1.28)	0.256
Discharge heart rate <60 bpm	0.91 (0.41 – 1.58)	0.347
Discharge systolic BP <100 mmHg	0.76 (0.23 – 0.98)	<0.001
Pleural effusion at discharge	0.81 (0.48 – 1.38)	0.455
Peripheral oedema at discharge	0.88 (0.38 – 1.56)	0.688
Hyperkalaemia	0.81 (0.21 – 1.35)	0.455
History of CKD	0.84 (0.64 – 1.01)	0.056
Worsening renal function during hospitalisation	0.54 (0.13 – 0.93)	<0.001
Inotropes/vasopressors use	0.71 (0.43 – 0.93)	<0.001
Peak daily dose of intravenous diuretic	0.89 (0.64 – 1.36)	0.122
Addition of thiazide-type diuretic or oral acetazolamide during hospitalisation	1.12 (0.68 – 1.46)	0.101
Length of hospital stay	1.06 (0.98 – 1.29)	0.199
**Sacubitril/valsartan**		
Sex (male)	1.03 (0.81 – 1.19)	0.689
Age (per year)	0.95 (0.87 – 0.97)	<0.001
LVEF (%)	1.14 (1.07 – 1.20)	<0.001
NYHA class III-IV	0.99 (0.90 – 1.15)	0.178
Discharge heart rate <60 bpm	0.87 (0.76 – 0.95)	0.007
Low BP at discharge*	0.56 (0.33 – 0.79)	<0.001
Pleural effusion at discharge	0.75 (0.55 – 0.96)	<0.001
Peripheral oedema at discharge	0.80 (0.72 – 0.95)	<0.001
Hyperkalaemia	0.77 (0.61 – 0.97)	0.008

History of CKD	0.82 (0.71 – 0.94)	0.006
Worsening renal function during hospitalisation	0.57 (0.38 – 0.88)	<0.001
Inotropes/vasopressors use	0.68 (0.51 – 0.88)	<0.001
Peak daily dose of intravenous diuretic	1.01 (0.88 – 1.13)	0.569
Addition of thiazide-type diuretic or oral acetazolamide during hospitalisation	1.13 (1.01 – 1.38)	0.002
Length of hospital stay	0.94 (0.89 – 0.97)	<0.001
**Beta-blockers**		
Sex (male)	1.02 (0.90 – 1.01)	0.567
Age (per year)	0.96 (0.91 – 0.98)	<0.001
LVEF (%)	1.01 (0.99 – 1.10)	0.189
NYHA class III-IV	1.18 (1.12 – 1.29)	0.002
Discharge heart rate <60 bpm	0.74 (0.63 – 0.91)	<0.001
Discharge systolic BP <100 mmHg	0.89 (0.71 – 0.97)	0.011
Pleural effusion at discharge	0.81 (0.72 – 0.95)	<0.001
Peripheral oedema at discharge	0.89 (0.72 – 1.00)	0.052
Hyperkalaemia	0.99 (0.87 – 1.21)	0.278
History of CKD	1.12 (0.98 – 1.21)	0.344
Worsening renal function during hospitalisation	0.82 (0.69 – 0.91)	<0.001
Inotropes/vasopressors use	0.68 (0.43 – 0.90)	<0.001
Peak daily dose of intravenous diuretic	0.98 (0.65 – 1.31)	0.899
Addition of thiazide-type diuretic or oral acetazolamide during hospitalisation	1.11 (0.96 – 1.27)	0.115
Length of hospital stay	0.95 (0.91 – 0.98)	<0.001
**MRA**		
Sex (male)	1.10 (0.99 – 1.16)	0.075
Age (per year)	0.94 (0.83 – 0.99)	0.003
LVEF (%)	1.06 (0.98 – 1.12)	0.233
NYHA class III-IV	0.90 (0.86 – 0.95)	0.001
Discharge heart rate <60 bpm	1.01 (0.86 – 1.21)	0.328
Discharge systolic BP <100 mmHg	0.96 (0.87 – 1.01)	0.655
Pleural effusion at discharge	0.88 (0.81 – 0.95)	<0.001
Peripheral oedema at discharge	0.91 (0.83 – 0.99)	0.038
Hyperkalaemia	0.45 (0.21 – 0.66)	<0.001
History of CKD	0.56 (0.23 – 0.70)	<0.001
Worsening renal function during hospitalisation	0.48 (0.28 – 0.68)	<0.001
Inotropes/vasopressors use	1.11 (0.98 – 1.32)	0.367
Peak daily dose of intravenous diuretic	0.85 (0.74 – 0.98)	0.027
Addition of thiazide-type diuretic or oral acetazolamide during hospitalisation	0.94 (0.78 – 1.23)	0.267
Length of hospital stay	0.99 (0.91 – 1.07)	0.118
**SGLT2I**		
Sex (male)	1.01 (0.94 – 1.37)	0.895
Age (per year)	0.98 (0.86 – 1.25)	0.788
LVEF (%)	1.03 (0.87 – 1.32)	0.876
NYHA class III-IV	0.96 (0.88 – 1.37)	0.455

Discharge heart rate <60 bpm	1.05 (0.89 – 1.16)	0.476
Discharge systolic BP <100 mmHg	0.95 (0.87 – 0.98)	<0.001
Pleural effusion at discharge	0.98 (0.91 – 1.16)	0.899
Peripheral oedema at discharge	0.97 (0.89 – 1.03)	0.787
Hyperkalaemia	0.96 (0.86 – 0.99)	0.011
History of CKD	0.79 (0.68 – 0.86)	<0.001
Worsening renal function during hospitalisation	0.52 (0.35 – 0.64)	<0.001
Inotropes/vasopressors use	1.04 (0.87 – 1.13)	0.285
Peak daily dose of intravenous diuretic	0.88 (0.76 – 1.07)	0.119
Addition of thiazide-type diuretic or oral acetazolamide during hospitalisation	1.05 (0.88 – 1.19)	0.371
Length of hospital stay	0.96 (0.92 – 0.99)	0.001
**Less than three key drug classes**		
Sex (male)	0.98 (0.91 – 1.12)	0.112
Age (per year)	1.07 (1.02 – 1.10)	0.001
LVEF (%)	0.87 (0.64 – 0.91)	<0.001
NYHA class III-IV	1.15 (1.10 – 1.25)	<0.001
Discharge heart rate <60 bpm	1.33 (1.22 – 1.56)	<0.001
Discharge systolic BP <100 mmHg	2.21 (1.88 – 2.78)	<0.001
Pleural effusion at discharge	1.23 (1.11 – 1.34)	<0.001
Peripheral oedema at discharge	1.16 (1.08 – 1.34)	<0.001
Hyperkalaemia	1.69 (1.23 – 2.00)	<0.001
History of CKD	2.11 (1.78 – 3.10)	<0.001
Worsening renal function during hospitalisation	2.78 (2.23 – 3.21)	<0.001
Inotropes/vasopressors use	1.15 (1.02 – 0.28)	0.031
Peak daily dose of intravenous diuretic	1.09 (1.00 – 1.14)	0.051
Addition of thiazide-type diuretic or oral acetazolamide during hospitalisation	0.86 (0.80 – 0.94)	0.005
Length of hospital stay	1.07 (1.02 – 1.18)	<0.001

ACEI – angiotensin-converting enzyme inhibitor, ARB – angiotensin receptor blocker, BP – blood pressure, CI – confidence interval, CKD -chronic kidney disease, LVEF – left ventricular ejection fraction, MRA – mineralocorticoid-receptor antagonist, NYHA – New York Heart Association, OR – odds ratio, SGLT2I – sodium-glucose cotransporter-2 inhibitor.
